# Serotonin/Dopamine Interactions in a Hyperactive Mouse: Reduced Serotonin Receptor 1B Activity Reverses Effects of Dopamine Transporter Knockout

**DOI:** 10.1371/journal.pone.0115009

**Published:** 2014-12-16

**Authors:** Frank Scott Hall, Ichiro Sora, René Hen, George R. Uhl

**Affiliations:** 1 Department of Pharmacology, College of Pharmacy and Pharmaceutical Sciences, University of Toledo, Toledo, Ohio, United States of America; 2 Molecular Neurobiology Branch, National Institute on Drug Abuse – Intramural Research Program, Baltimore, Maryland, United States of America; 3 Kobe University Graduate School of Medicine, Kobe, Japan; 4 Departments of Pharmacology, Neuroscience and Pharmacology, Columbia University, New York, New York, United States of America; Division of Integrative Neuroscience, The New York State Psychiatric Institute, New York, New York, United States of America; Tokyo Metropolitan Institute of Medical Science, Japan

## Abstract

Knockout (KO) mice that lack the dopamine transporter (SL6A3; DAT) display increased locomotion that can be attenuated, under some circumstances, by administration of drugs that normally produce psychostimulant-like effects, such as amphetamine and methylphenidate. These results have led to suggestions that DAT KO mice may model features of attention deficit hyperactivity disorder (ADHD) and that these drugs may act upon serotonin (5-HT) systems to produce these unusual locomotor decreasing effects. Evidence from patterns of brain expression and initial pharmacologic studies led us to use genetic and pharmacologic approaches to examine the influence of altered 5-HT_1B_ receptor activity on hyperactivity in DAT KO mice. Heterozygous 5-HT_1B_ KO and pharmacologic 5-HT_1B_ antagonism both attenuated locomotor hyperactivity in DAT KO mice. Furthermore, DAT KO mice with reduced, but not eliminated, 5-HT_1B_ receptor expression regained cocaine-stimulated locomotion, which was absent in DAT KO mice with normal levels of 5-HT_1B_ receptor expression. Further experiments demonstrated that the degree of habituation to the testing apparatus determined whether cocaine had no effect on locomotion in DAT KO or reduced locomotion, helping to resolve differences among prior reports. These findings of complementation of the locomotor effects of DAT KO by reducing 5-HT_1B_ receptor activity underscore roles for interactions between specific 5-HT receptors and dopamine (DA) systems in basal and cocaine-stimulated locomotion and support evaluation of 5-HT_1B_ antagonists as potential, non-stimulant ADHD therapeutics.

## Introduction

Dopamine (DA) systems have long been implicated in the control of basal [1], [2] and stimulant-induced locomotion [3]–[5]. Activity of the dopamine transporter (SLC6A3, DAT) is a major determinant of DA compartmentalization, modulating the dynamic temporal profiles of intracellular, synaptic, and extrasynaptic DA levels [6], [7]. The elimination of DAT expression in DAT KO mice leads to profound changes in DA release dynamics [8], elevated basal levels of extracellular DA [8], [9] and profound locomotor hyperactivity [10], [11]. Cocaine-induced locomotion is also eliminated in DAT KO mice [10], [12]. Indeed, under some conditions, decreased locomotion is observed in DAT KO mice after administration of cocaine or other psychostimulant drugs [13], mimicking the therapeutic effects that stimulants provide for many individuals with attention deficit hyperactivity disorder (ADHD) [14], [15]. DAT KO mice also have deficits in pre-pulse inhibition of acoustic startle (PPI) [16] that are reversed by psychostimulant drugs [17]. Similar to the effects of these drugs on locomotion, although they improve PPI in DAT KO mice, they produce impairments in wildtype (WT) mice.

This pattern of effects of psychostimulant drugs on locomotor behavior and PPI have led to suggestions that DAT KO mice may serve as a model of ADHD [18], [19]. Effects of psychostimulant drugs on serotonin (5-HT) function were initially suggested to be involved in the amelioration of hyperactivity in DAT KO mice by psychostimulant drugs based on the effects of other serotonergic agents in this model [13]. 5-HT systems, via interactions with DA systems, have been implicated in modulation of both basal and psychostimulant-induced locomotion for some time [20]–[22]. 5-HT systems that act through 5-HT_1B_ receptors are especially promising candidates to contribute to these direct and interactive influences on locomotor behavior based upon their placement in this circuitry [23]–[25]. High levels of 5-HT_1B_ receptors are expressed by striatonigral neurons that provide direct pathway feedback to ventral midbrain dopaminergic neurons that are heavily implicated in motor control [26]–[29]. 5-HT can facilitate DA release [30], [31] in ways that involve 5-HT_1B_ receptors [32], although other 5-HT receptor subtypes also modulate DA release and neuronal activity [33]–[35]. Combined deletion of DAT and the serotonin transporter (SERT) to produce DAT/SERT double KO mice results in even higher basal locomotion than that displayed by DAT deletion alone and 5-HT_1B_ receptor levels are reduced in the substantia nigra of these mice [11]. 5-HT_1B_ receptor KO mice display increased locomotor responses to cocaine [36], while their levels of basal locomotion are similar to those of wildtype (WT) mice [37], [38].

To elaborate further the role of 5-HT_1B_ receptors in locomotor behavior, and in the effects of DAT KO on locomotor behavior, genetic and pharmacologic approaches were used in the present experiments. There was substantial convergence between the results from studies of lifelong deletion of half of the normal quantity of 5-HT_1B_ receptors and acute blockade of 5-HT_1B_ receptors in these experiments. Furthermore, examination of basal and cocaine-stimulated locomotor activity after both long and short periods of habituation identified this factor, which differed in previous studies in DAT KO mice, as the critical factor determining whether reductions in locomotion were observed after cocaine treatment in DAT KO mice. The implications of the prominent role for 5-HT_1B_ receptors in serotonin-dopamine interactions in DAT KO mice identified in these experiments is considered for the potential to develop non-psychostimulant therapeutics the treatment of ADHD.

## Results

### Experiment 1: Basal and Cocaine-induced Locomotor Activity after an Extended Period of Habituation

**Figure 1:**
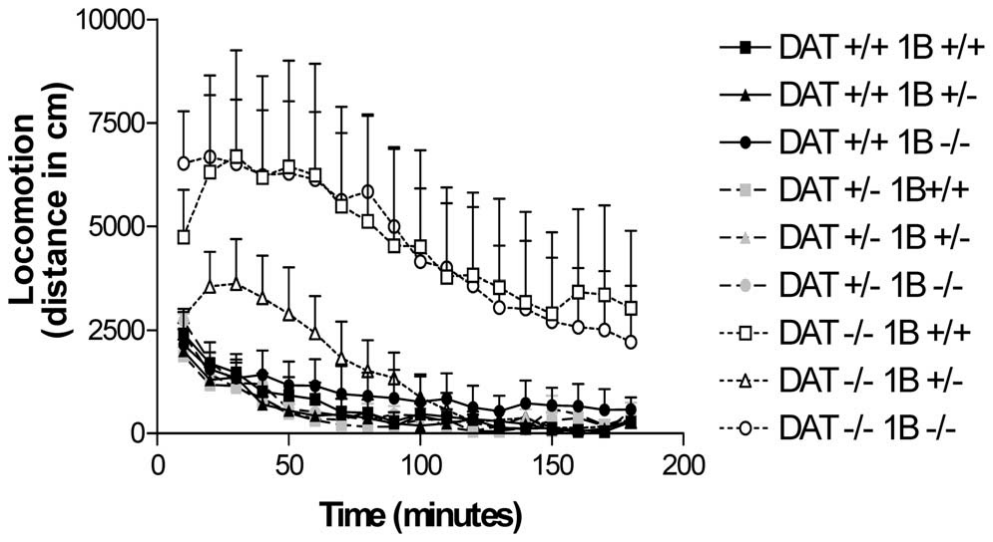
Experiment 1: Time-course of baseline locomotor activity in combined DAT/5-HT1B KO mice: long habituation session. Time-course of locomotor activity during the first habituation session in DAT/5-HT1B KO mice (9 genotypes: DAT +/+5-HT1B +/+, DAT −/−5-HT1B +/+, DAT +/+5-HT1B −/−, DAT +/−5-HT1B −/−, DAT −/−5-HT1B +/−, DAT −/−5-HT1B −/−) expressed in terms of total distance traveled. Data are represented as mean ± SEM.

During the three hour period of habituation prior to cocaine administration locomotor activity was greater in DAT −/− mice than in DAT +/− or DAT +/+ mice (Fig. 1; DAT GENOTYPE: F [2], [81] = 11.4, p<0.0001). In combined knockout mice, the effect of DAT KO depended strikingly on 5-HT_1B_ genotype. The basal hyperactivity found in DAT −/−5-HT_1B_ +/+ mice was reduced sharply, to nearly normal levels, in DAT −/−5-HT_1B_ +/− mice that have reduced 5-HT_1B_ receptor expression. This effect appears to require some 5-HT_1B_ expression, however, since the genetically complementing influence of reducing 5-HT_1B_ expression was not observed in mice with complete elimination of both DAT and 5-HT_1B._ Locomotion in DAT −/−5-HT_1B_ −/− mice was thus similar to the levels of hyperactivity noted in the DAT −/−5-HT_1B_ +/+ mice.

**Figure 2:**
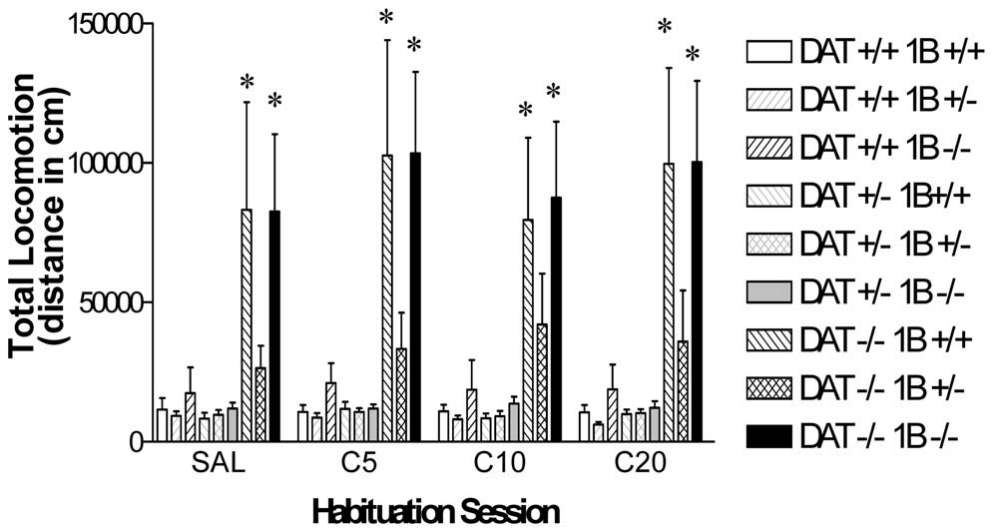
Experiment 1: Baseline locomotor activity in well habituated combined DAT/5-HT1B KO mice. Total locomotor activity in DAT/5-HT1B KO mice (9 genotypes: DAT +/+5-HT1B +/+, DAT −/−5-HT1B +/+, DAT +/+5-HT1B −/−, DAT +/−5-HT1B −/−, DAT −/−5-HT1B +/−, DAT −/−5-HT1B −/−) expressed in terms of summed distance traveled during all of the 4 habituation sessions prior to drug (5, 10 and 20 mg/kg IP cocaine; C5, C10 and C20 respectively) or saline (SAL) injections. *Significant difference from DAT +/+5-HT1B +/+ mice based on Scheffe’s post hoc comparison (p<0.05). Data are represented as mean ± SEM.

There were no significant differences in between-session habituation between mice of the differing genotypes. Differences in basal locomotion observed in the first session continued to be observed in later habituation sessions that preceded dose-response testing later in this experiment. Locomotor activity scores that were summed for subsequent habituation sessions revealed group effects that were virtually identical to the locomotion observed during the initial habituation session (Fig. 2). Data in Fig. 2 documents the persistence of the lower activity of DAT −/−5-HT_1B_ +/− mice when compared to either DAT −/−5-HT_1B_ +/+ or DAT −/−5-HT_1B_ −/− mice. The main effect of DAT GENOTYPE was highly significant (F [2], [81] = 14.9, p<0.0001). *Post hoc* examination of the DAT GENOTYPE effect demonstrated that these effects were dependent on the 5-HT_1B_ receptor genotype. Scheffe’s comparisons for each DAT −/− genotype group versus DAT +/+5-HT_1B_ +/+ mice found significantly greater locomotion in DAT −/−5-HT_1B_ +/+ mice and DAT −/−5-HT_1B_ −/− mice, but not in DAT −/−5-HT_1B_ +/− mice, for all habituation sessions compared to DAT +/+5-HT_1B_ +/+ mice (Fig. 2). By contrast, due to the inverse 5-HT_1B_ gene dose-response relationship, neither the main effect of 5-HT_1B_ GENOTYPE (F [2], [81] = 1.8, ns) nor the DAT GENOTYPE×5-HT_1B_ GENOTYPE interaction (F [4], [81] = 1.3, ns) reached statistical significance.

**Figure 3:**
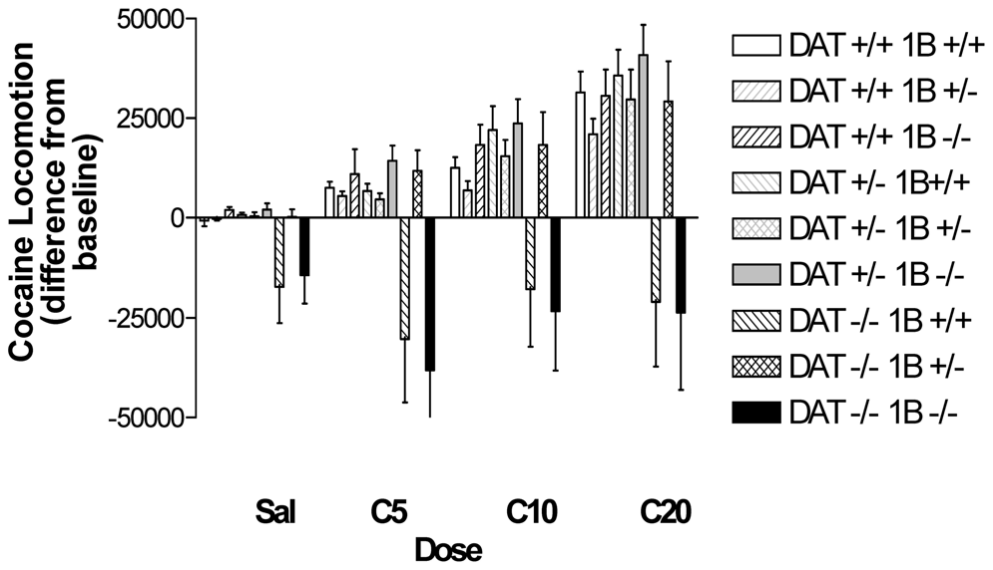
Experiment 1: Cocaine-induced locomotion in well habituated DAT/5-HT1B KO mice. Cocaine-induced locomotor activity in DAT/5-HT1B KO mice is expressed as absolute difference s from baseline, preinjection values. Post hoc analysis by 1 way ANOVA for COCAINE concentration revealed significant effects of cocaine for all genotypes except DAT −/−5-HT1B +/+ and DAT −/−5-HT1B −/−. Data are represented as mean ± SEM.

Locomotor responses to saline or cocaine injection are presented in Fig. 3. The differences between locomotion following injection and the summed locomotion in the two hours of habituation prior to drug injection are represented to correct for differences in baseline activity. Saline administration did not increase locomotion. Activity of mice of most genotypes, including DAT −/−5-HT_1B_ +/− mice, was minimal after saline injections. By contrast, the locomotor activity of DAT −/−5-HT_1B_ +/+ and DAT −/−5-HT_1B_ −/− mice continued to decrease after saline injections; these mice appear to experience continued habituation from the higher levels of locomotor activity that they display at the beginning of the test period. As previously reported [12], DAT −/−5-HT_1B_ +/+ mice exhibited no locomotor stimulant responses to cocaine. These differences from the substantial increases observed in DAT +/+5-HT_1B_ +/+ and DAT +/−5-HT_1B_ +/+ mice resulted in an overall significant effect of DAT GENOTYPE (F [2], [81] = 14.9, p<0.0001). Failure to observe increases in DAT −/−5-HT_1B_ +/+ mice did not appear to be due to ceiling effects. Habituation had reduced locomotion to approximately half of initial levels when cocaine was administered (see Fig. 1).

Cocaine administration significantly increased locomotion, with maximal increases at 20 mg/kg (Fig. 3; DOSE: F [3,243] = 36.6, p<0.0001), although increases were not apparent in mice of all genotypes. *Post hoc* evaluations of the effect of DOSE (by 1-way ANOVA) in each genotype revealed a significant effect of DOSE in DAT −/−5-HT_1B_ +/− mice that was not observed in DAT −/−5-HT_1B_ +/+ or DAT −/−5-HT_1B_ −/− mice. While deletion of a single copy of the 5-HT_1B_ receptor gene in DAT −/−5-HT_1B_ +/− mice restored the ability of cocaine to increase locomotion, some 5-HT_1B_ expression appears necessary to normalize responses to cocaine in DAT −/− mice. Significant effects of cocaine DOSE were observed in mice of all other genotypes (p<0.01). The interaction of cocaine DOSE and DAT GENOTYPE was not significant in the ANOVA, however.

### Experiment 2: Cocaine-induced Locomotor Activity after a Brief Period of Habituation

**Figure 4:**
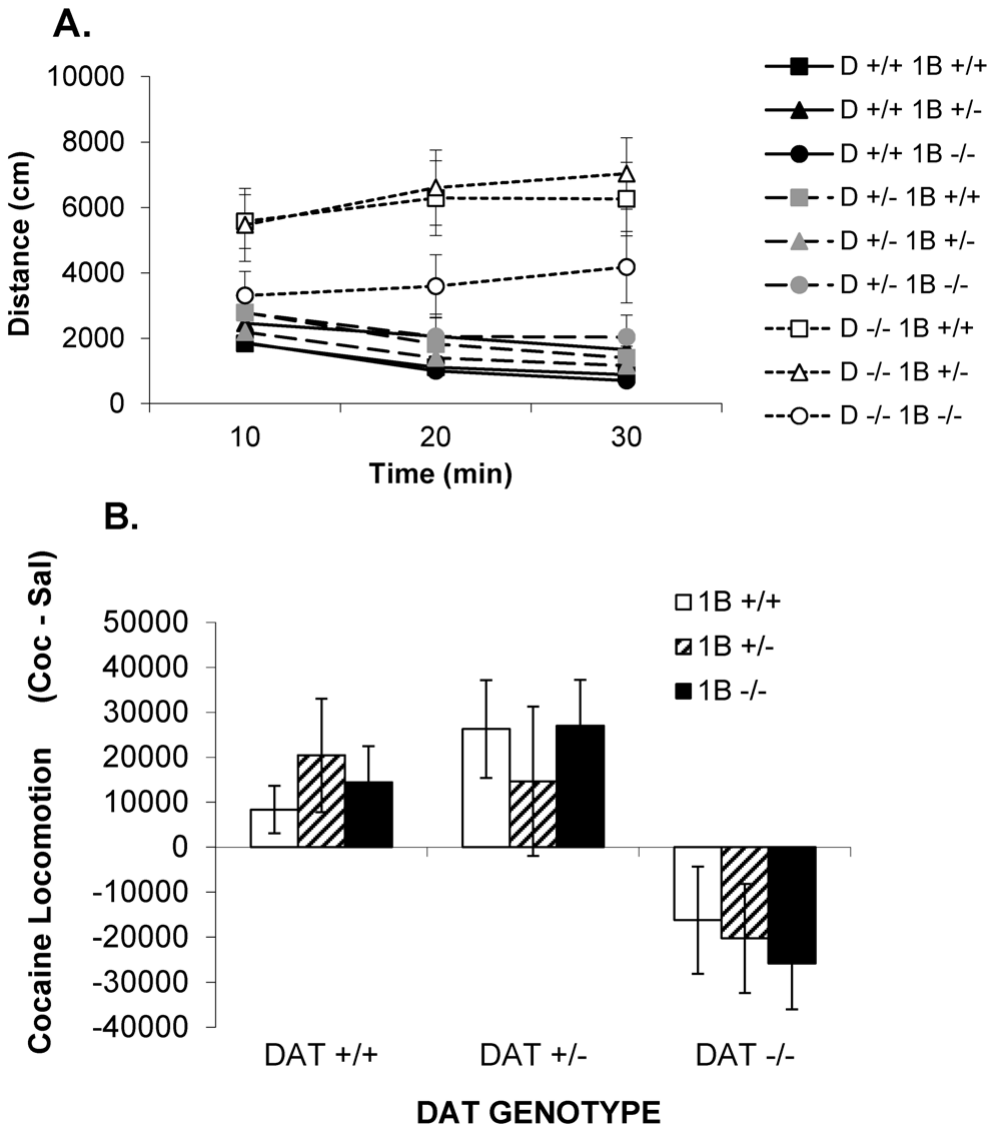
Experiment 2: Locomotor activity in combined DAT/5-HT1B KO mice: short habituation sessions. (A) Time-course of locomotor activity during the first habituation session in DAT/5-HT1B KO mice (9 genotypes: DAT +/+5-HT1B +/+, DAT −/−5-HT1B +/+, DAT +/+5-HT1B −/−, DAT +/−5-HT1B −/−, DAT −/−5-HT1B +/−, DAT −/−5-HT1B −/−) expressed in terms of total distance traveled. (B) Cocaine-induced locomotor activity in DAT/5-HT1B KO mice expressed as absolute difference from saline (total activity after cocaine injection – total activity after saline injection). Data are represented as mean ± SEM.

During 30 minute short habituation sessions, DAT −/− mice were significantly more active than mice of all other genotypes (DAT GENOTYPE: F [2,97] = 30.6, p<0.0001; Fig. 4A). During this shorter habituation period there was no significant complementation observed between DAT and 5-HT_1B_ genotypes (DAT GENOTYPE×5-HT_1B_ GENOTYPE: F [4,97] = 1.9, NS), although the same overall pattern was observed as in Experiment 1. Baseline differences in locomotor activity did not equalize over 30 min habituation periods. Effects of cocaine on locomotion were thus evaluated as the difference between activity after cocaine administration and activity after saline administration (Fig. 4B).

There was a significant effect of DAT GENOTYPE on the locomotor stimulant effects of cocaine (Fig. 4B; F [2,97] = 11.4, p<0.0001). However, there were no significant effects of either 5-HT_1B_ GENOTYPE (F [2,97] = 0.1, ns) nor interaction between DAT GENOTYPE and 5-HT_1B_ GENOTYPE (F [4,97] = 0.4, ns). The significant effect of DAT GENOTYPE reflects the large increases in locomotion produced by cocaine administration after this short period of habituation period in DAT +/+ mice and DAT +/− mice, but not DAT −/− mice of any 5-HT_1B_ genotype. Under these experimental conditions cocaine reduced locomotion below levels observed after saline injection alone in DAT −/− mice. 5-HT_1B_ genotype failed to produce any significant effects on locomotor responses to cocaine administered after this short period of habituation.

**Figure 5:**
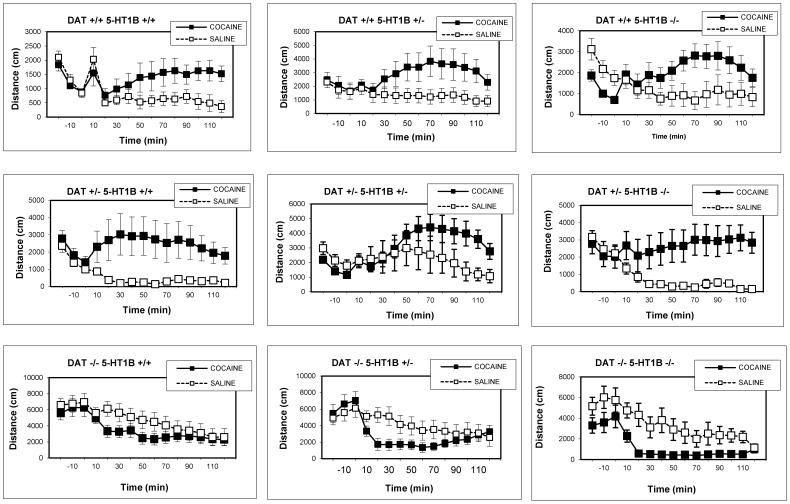
Experiment 2: Time-course of locomotor activity in DAT/5-HT1B KO mice, short habituation sessions. Time-course of saline and cocaine-induced locomotor activity in DAT/5-HT1B KO mice, represented separately for each genotype. Cocaine increased locomotion in all DAT +/+ and DAT +/− genotypes (p<0.01), and reduced locomotion in all DAT −/− genotypes (p<0.01) except DAT −/−5-HT1B, which was confirmed by individual ANOVA comparing saline and cocaine locomotion for each genotype. Data are represented as mean ± SEM.

Analyses of the time course of locomotor activity for each genotype separately (Fig. 5) revealed increased locomotor activity after cocaine in all DAT +/+ and DAT +/− genotypes, regardless of 5-HT_1B_ genotype. There were significant DRUG×TIME interactions (p<0.01) for all DAT +/+ and DAT +/− genotypes. For DAT −/− genotypes cocaine decreased activity. This effect achieved significance in DAT −/−5-HT_1B_ +/− and DAT −/−5-HT_1B_ −/− mice, reflected by significant DRUG×TIME interactions (p<0.01), but not in DAT −/−5-HT_1B_ +/+ mice. 5-HT_1B_ KO thus appears to slightly enhance the locomotion-reducing effects when cocaine is administered after a short period of habituation.

### Experiment 3: The effects of the 5-HT antagonist SB 224289 on locomotor activity in DAT −/− and DAT +/+ mice

**Figure 6:**
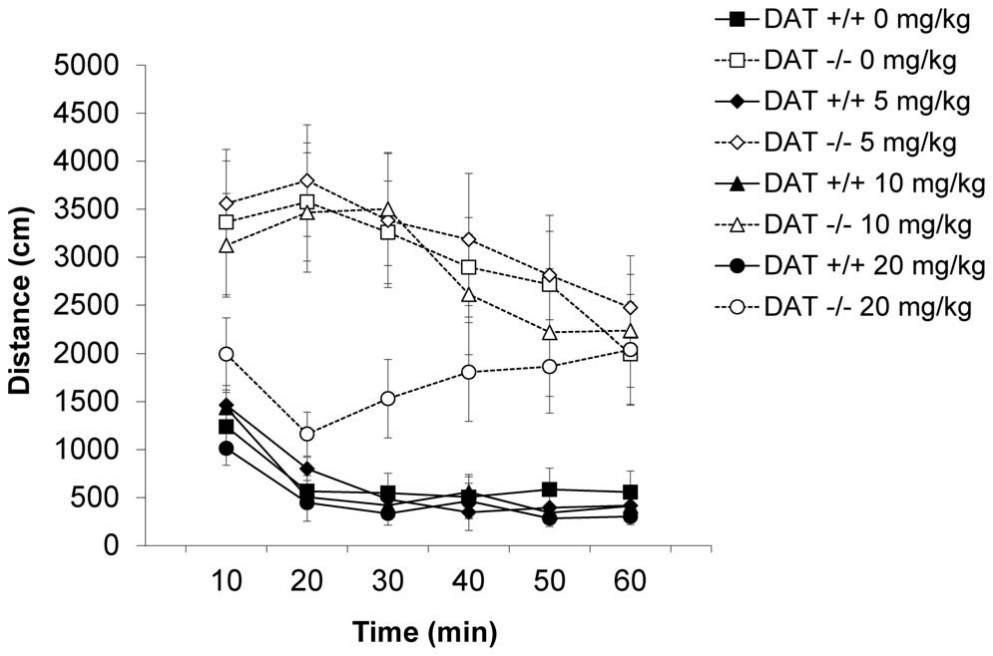
Experiment 3: The effects of the 5-HT antagonist SB 224289 on locomotor activity in DAT KO and WT mice. Time-course locomotor activity in DAT −/− and DAT +/+ mice after injection with 0, 5, 10, or 20 mg/kg SB 224289. Data are represented as mean ± the SEM.

In this experiment locomotor activity was again elevated in DAT −/− mice compared to DAT +/+ mice (Fig. 6; DAT GENOTYPE: F [1], [18] = 30.6, p<0.0001). The high dose of SB 224289 significantly reduced locomotor activity in the hyperactive DAT −/− mice (DRUG: F [3], [54] = 4.0, p<0.02) but not in DAT +/+ mice. There was thus a significant GENOTYPE×DRUG×TIME interaction (F [15, 270] = 2.2, p<0.01). *Post hoc* comparisons of the effect of SB 224289 in each genotype separately confirmed these observations. SB 224289 had no effect on locomotor activity in DAT +/+ mice (DOSE: F [3], [27] = 0.5, ns; DOSE×TIME: F [15, 135] = 1.1, ns), but the 5-HT_1B_ antagonist significantly reduced activity in DAT −/− mice (DOSE: F [3], [27] = 3.5, p<0.03; DOSE×TIME: F [15, 135] = 2.3, p<0.01).

## Discussion

The present data provide striking evidence that: 1) either chronic genetic or acute pharmacologic reduction in 5-HT_1B_ receptor function can reduce locomotor impairments in DAT KO mice; 2) cocaine-induced locomotor activation can be obtained in the absence of DAT when 5-HT_1B_ receptor function is reduced; and 3) psychostimulant drugs can decrease locomotion in DAT KO mice under conditions in which only modest habituation occurs prior to drug administration. The current data confirm and extend previous findings of: a) locomotor hyperactivity and elimination of cocaine-stimulated locomotion in DAT knockout mice [10], [12], and b) cocaine-induced reductions in locomotor activity when administered to mice after short habituation periods [13]. We discuss these observations in the light of evidence for involvement of DAT and different 5-HT_1B_ receptor subpopulations in basal- and cocaine-stimulated locomotion, and of their implications for understanding this model of ADHD.

### Genetic complementation and pharmacological confirmation

DAT deletion produces baseline hyperactivity but prevents further cocaine-induced increases in activity. We show here that the effects of DAT deletion on basal and cocaine-induced locomotion were reversed when a heterozygous 5-HT_1B_ receptor KO was combined with a homozygous DAT deletion. This evidence for classical genetic complementation, reversal of influences of one genetic change when another is added, provides strong evidence for the importance of 5-HT systems that involve 5-HT_1B_ receptors in the locomotor phenotypes observed in DAT KO mice. The pharmacological results of acute administration of the 5-HT_1B_ antagonist SB 224289 buttress these genetic findings, and make it less likely that this result depends on adaptations to chronic 5-HT_1B_ depletions in the heterozygous KO mice.

### 5-HT_1B_ gene dose response relationship and comparisons previous findings

By contrast to the results in heterozygous 5-HT_1B_ KO mice, complete deletion of the 5-HT_1B_ receptor in homozygous 5-HT_1B_ KO mice does not alter the locomotor effects of DAT gene deletion. These observations support an inverse gene-dose relationship and a requirement for some level of 5-HT_1B_ receptor function to normalize locomotor behavior in homozygous DAT −/− mice. This complex gene-dose relationship for homozygous DAT KO mice appears to be accompanied by differences in dose-response relationships for 5-HT_1B_ antagonists in modulating locomotion in different genetic and behavioral settings. In the present work, mice with wildtype DAT genetic backgrounds displayed no significant differences in locomotion after either genetic 5-HT_1B_ manipulations or 5-HT_1B_ antagonist treatment. Acute 5-HT_1B_ antagonist treatment also exerted only minimal effects on locomotion in WT mice in another study, although 5-HT_1B_ antagonism restored methamphetamine-induced locomotor sensitization in serotonin transporter (SLC6A4; SERT) KO mice in that study [39]. These divergent effects of 5-HT_1B_ manipulations on locomotor activity in DAT KO and SERT KO mice, compared to WT mice, bear some similarity to the divergent effects on reinforcement in animals with different experimental histories. 5-HT_1B_ agonists have been shown to enhance cocaine reinforcement but decrease cocaine-seeking in a reinstatement model, when they are administered following different periods of cocaine self-administration and withdrawal [40], [41].

An interesting point here is the lack of effect of 5-HT_1B_ manipulations alone. Our results differ from those reported by Castanon et al (2000), who found that 5-HT_1B_ KO increased cocaine-induced locomotion while 5-HT_1B_ antagonists reduced cocaine-induced locomotion. These differences might be due to differences in the state of the mice tested, since increases in basal or stimulant-induced locomotor activity are likely to depend on dopaminergic cell firing rates that can be altered substantially by habituation to the environment [42], as was shown in the present experiments when habituation prior to drug injection was specifically manipulated. Genetic background might also play a role in these differences. Castanon et al (2000) used an inbred 129/Sv 5-HT_1B_ KO strain for the genetic study, but C57Bl/6J mice for the pharmacological study. Genetic variation across strains, or genetic manipulations that cause perturbations in function throughout this circuitry such as DAT and SERT, would appear to alter the consequences of 5-HT_1B_ receptor manipulations.

### Adaptation in KO mice versus acute differences in psychostimulant responses

Studies of the adaptations to the chronic loss of DAT function in DAT KO mice have centered on DA receptor dynamics [8], [10], [43]–[45]. Our current results should add impetus to studies of 5-HT systems and DA-5-HT interactions in these animals as well [9], [13], [43]. We have previously reported reduced 5-HT_1B_ receptor expression in mice with combined DAT −/− SERT −/− gene deletions [11], but have not identified changes in brain 5-HT_1B_ mRNA expression in DAT KO mice (Li, Hall and Uhl, unpublished findings). More subtle region- or cell-specific changes are still possible, as are adaptations at other (e.g. circuitry) levels (see below).

Roles for adaptations to the removal of 5-HT_1B_ receptors also remain possible, although the fit between data from 5-HT_1B_ receptor knockdown (e.g. heterozygous KO mice) and acute antagonist treatments is consistent with direct roles of 5-HT_1B_ systems/acute alterations in many of the effects observed here. Brain changes reported in homozygous 5-HT_1B_ KO mice do include reduced 5-HT levels, increased DA levels, and enhanced cocaine-induced DA release in nucleus accumbens [46]; reduced striatal SERT binding [47]; reductions in 5-HT_2C_ receptor function [48]; reduced cocaine-induced c-fos elevation [49]; and changes in several other receptor systems [50]. The degree to which these changes occur in heterozygous 5-HT_1B_ KO mice, if at all, is not known however, which adds to the likelihood that the effects of heterozygous 5-HT_1B_ deletion are the result of the reduced 5-HT_1B_ receptor numbers, not other chronic adaptations. The gene-dose dependence of the results that we report here, and their convergence with the results of acute pharmacological interventions, both focus attention on mechanisms that require at least some 5-HT_1B_ receptors, and away from mechanisms that are engaged by the total absence of 5-HT_1B_ receptors. Observations that mice without any functional copies of either DAT or 5-HT_1B_ lack any significant cocaine-induced locomotion support the idea that normal 5-HT_1B_ mechanisms are involved in cocaine-induced locomotor stimulation in DAT KO mice and mechanisms that reduce locomotion in WT mice.

### Dopamine, serotonin and locomotor control

DA systems are widely implicated in locomotor control by data from DA lesion, antagonist and structure-activity studies [51]–[56]. Initial results in DAT KO mice supported the longstanding view that DA is the primary mediator of locomotor behavior and that cocaine is a locomotor stimulant due to its ability to elevate DA levels by blocking DAT. Current results demonstrating restoration of cocaine-induced locomotion in the absence of DAT in DAT −/−5-HT_1B_ +/− mice add to other evidence that this view is oversimplified. Support for 5-HT_1B_ receptors as plausible sites for serotonergic effects on basal- and cocaine-modulated locomotor activity comes from increased locomotion induced by the 5-HT_1B_ receptor agonist RU24969 [57] that can be blocked by the 5-HT_1B_ receptor antagonist GR127935 [58] and 5-HT_1B_ agonist treatment can potentiate nucleus accumbens dopamine release in wildtype mice [59]. This pharmacological data alone might suggest that 5-HT_1B_ receptors might have a role in ADHD.

### Relevance to ADHD and ADHD symptomatology

DAT KO mice have been proposed to constitute an animal model of ADHD based on the behavioral phenotypes observed in these mice and responses to drugs that treat ADHD [13], [18]. This is consistent with transmission disequilibrium studies that have associated human DAT genomic markers with ADHD in several, though not all, clinical samples [60]–[63], although certainly none of those studies identified a homozygous DAT KO in humans. Nonetheless, the decreased locomotion noted in stimulant-treated DAT −/− mice under some circumstances [13] has been suggested to parallel the “calming” effects of psychostimulants in hyperactive children [14], [15]. The dependence of these effects in DAT −/− mice on the degree of environmental stimulation in the current habituation studies is consistent with reports in ADHD as well. Similar to these effects on DAT hyperactivity, while administration of psychostimulant drugs to WT mice impairs PPI, the deficits in PPI found in DAT KO mice are reversed by administration of psychostimulant drugs that treat ADHD [17], the selective norepinephrine reuptake blocker nisoxetine [17], and nicotine [64]. Much of this data is consistent with the rate dependency theory of drug action in ADHD [65], which has gained support from both clinical and brain imagining studies [66], [67].

Genetic studies in humans have associated the *htr1B* gene with ADHD in some studies [68]–[71]. Such associations have not been found in all studies, but suggestions of gene-gene interactions for ADHD susceptibility and parent of origin effects [72] may account for some of these negative findings. Furthermore, those authors suggested that epigenetic regulation of *htr1B* may also be involved. That expression of the 5-HT_1B_ receptor may affect ADHD is also suggested by observations that genetic differences in the regulation of *htr1B* gene expression by the microRNA miR96 are also associated with ADHD [73]. It remains to be seen how genetic differences in this gene may interact with other genetic, or non-genetic, differences in DAT function in humans. The present results certainly suggest that this possibility should be investigated.

### Potential circuitry underlying DAT-5HT_1B_ interactions in DAT KO mice

Circuits connecting midbrain to striatum include ventral midbrain dopaminergic neurons that project to striatum, accumbens and cortex that receive reciprocal direct pathway inputs from striatofugal fibers [24]. There are substantial serotonergic inputs to these regions, including high concentrations of 5-HT_1B_ receptors on direct pathway/striatofugal connections from striatum to substantia nigra and ventral tegmental area neurons [23], [25]. Although it is not possible to exclude roles for other 5-HT_1B_ receptor populations based on the present results, it is tempting to focus on the direct pathway for explanations for many of the effects of reduced 5-HT_1B_ expression in DAT KO mice. 5-HT_1B_ receptors in the prefrontal cortex certainly modulate other behaviors [74], so it is possible that receptors in that brain region influence behavior in DAT KO mice, but the preponderance of evidence for 5-HT_1B_ influences on locomotion involve receptors in other brain regions. It thus appears most likely that the locus of 5-HT_1B_ effects is likely to be downstream from altered corticostriatal projections in DAT KO mice [75].

Accumulating evidence in DAT KO mice has indicated that the prefrontal cortex is the locus of psychostimulant drug effects in this model. Because of differences in the distribution of DAT in DA terminal regions, the consequences of DAT deletion on release dynamics in these regions are quite different. This can be observed in terms of the profound effect of DAT KO on basal DA levels in the striatum but rather minimal effects in the prefrontal cortex [9]. This imbalance in DA levels appears to be further associated with alterations in corticostriatal afferents and downstream circuitry [75], [76]. Subtle cognitive deficits have been found in DAT KO mice that are associated with changes in brain derived neurotrophic factor in the prefrontal cortex [77]. A potential model of aspects of impaired decision-making in ADHD, the cliff avoidance reaction, is also impaired in DAT KO mice and these deficits can also be reversed by drugs that treat ADHD [78]. One consequence of these changes is the impairment of PPI in these mice [16], which is ameliorated by administration of psychostimulant drugs or selective norepinephrine transporter (NET) blockers [17], the later finding suggesting that psychostimulant drugs produce these actions by blocking NET. Interestingly, in manner similar to effects of psychostimulants on locomotor activity, although psychostimulants normalize PPI in DAT KO mice, they produce impairments in wildtype mice. Furthermore, the locus of these effects is in the prefrontal cortex based on intracerebral injection experiments [75]. Nicotine, used as a form of self-treatment for attentional deficits in ADHD [79], also acts in the prefrontal cortex to ameliorate PPI deficits in DAT KO mice [64]. Although the role of 5-HT_1B_ receptors has not been specifically investigated in DAT KO mice using the PPI model, serotonergic mechanisms were suggested to be important in the amelioration of hyperactivity in DAT KO mice by psychostimulants [13], as well as in the mechanism of action of nicotine in ameliorating PPI deficits [64]. This certainly warrants further investigation.

### Roles for behavioral state

Previous studies in two strains of DAT KO mice produced separately in different laboratories found that DAT KO can eliminate the locomotor stimulant effects of cocaine [10]–[12], but also that cocaine can reduce locomotor activity in DAT KO mice [13]. The major difference in this later study from the previous studies appeared to be the period of habituation prior to drug injection. Experiments 1 and 2 therefore confirm both types of psychostimulant effects in the same strain of DAT KO mice under similar experimental conditions: elimination of cocaine-induced increase in locomotor activity in Experiment 1 and cocaine-induced reductions in locomotion in Experiment 2. 5-HT_1B_ gene deletion thus has effects on basal and cocaine-stimulated locomotion that depend on DAT genotype and on the degree of habituation to the testing environment. Constitutive 5-HT_1B_ deletion alone does not affect basal or cocaine-stimulated locomotion in DAT +/+ mice under the conditions used here. Furthermore, in Experiment 3 the effect of the 5-HT_1B_ antagonist SB 224289 on locomotion was observed only in DAT −/−, and not in DAT +/+ mice. Taken together with effects of habituation (comparing Experiments 1 and 2), we can conclude that 5-HT_1B_ manipulations alter locomotion most prominently under conditions of high locomotor activity, e.g. with little habituation to the environment, or under conditions that otherwise elevate locomotion, such as DAT deletion. As discussed above, other data supports the idea that 5-HT_1B_ manipulations can increase or decrease DA mediated behavior under different conditions. Interestingly, it has also been reported 5-HT_1B_ agonists have biphasic effects on locomotion at different time periods after withdrawal from chronic cocaine self-administration [80].

### Conclusions

Observations that deletion of one copy of the 5-HT_1B_ receptor gene restores cocaine-induced locomotion in DAT −/− mice appears to rule out the simplest form of the “DAT is it” hypothesis of cocaine-induced locomotion, just as finding at least some retained cocaine reward in DAT KO mice ruled out the simple “DAT is it” hypothesis of cocaine reinforcement [12], [81]. This genetic complementation also supports at least some 5-HT_1B_-dependence of the development or expression of some of the adaptive processes found in DAT KO mice, including those that contribute to basal hyperactivity and to elimination of cocaine-stimulated locomotion [10]–[12]. The influence of 5-HT receptor activation on DA function is quite complex however, as indicated by the observation that 5-HT_1B_ antagonist treatment reduces locomotor sensitization to methamphetamine in WT mice but restores sensitization in SERT KO mice [39]. This finding, among others, makes it apparent that different 5-HT_1B_ receptor populations can influence locomotor behavior in different ways, and that the overall effect of 5-HT_1B_ receptor agonism or antagonism can be quite different under different circumstances.

The current data document how adaptations to lifelong absence of DAT can involve non-dopaminergic systems in addition to the well-documented alterations in dopaminergic parameters in DAT KO mice [8], [45]. Some of the adaptations to shorter-term blockade of DAT by psychostimulants may parallel these consequences of lifelong DAT deletion. Further studies that elucidate the roles of 5-HT_1B_ autoreceptors located on raphé neurons, 5-HT_1B_ receptors on striatonigral cells, and 5-HT_1B_ receptors located on other neurons in locomotor circuits will clarify the multiple roles of 5-HT_1B_ receptors in these different states. Such studies have helped to identify particular 5-HT_1B_ receptor populations involved in different aspects of cocaine reinforcement and reinstatement [82]. The current results thus seem to warrant increased consideration of 5-HT_1B_-dependent mechanisms in normal locomotor control, locomotor modulation by acute psychostimulant administration and variations in motor control due to genetic and/or developmental variations in dopaminergic systems that may be associated with ADHD.

## Materials and Methods

### Subjects

DAT [12] and 5-HT_1B_ receptor [83] KO mice were used to generate combined DAT/5-HT_1B_ KO mice by crosses of these lines to produce double heterozygous KO mice in the F1 generation, and subsequent double heterozygous crosses that resulted in all combinations of genotypes, including double homozygous KO mice, beginning in the F2 generation. Mice were of a mixed genetic background, C57BL/6J and 129/Sv, derived from each of the original KO strains. Mice were tested at 12–18 weeks of age. The number of subjects per condition in each particular experiment is listed below. Each genotype group consisted of equal numbers of males and female mice, but this was not included in the analysis and the group sizes are not listed separately by sex below. The majority of mice used in these studies were products of double heterozygous crosses, however, because of the rarity of double KO and double WT mice under these conditions, and the increased mortality that has been noted in DAT KO mice previously, an additional breeding strategy was used to produce a sufficient number of subjects; this involved crosses of DAT+/−5-HT_1B_ +/+, DAT +/+5-HT_1B_ +/−, and DAT +/−5-HT_1B_ −/− mice. All of these breeder mice were derived from double heterozygous crosses. Crosses of all genotypes produced similar initial litter sizes and expected genotype distributions (given the increased DAT mortality). Those crosses that produced higher numbers of DAT −/− mice than double heterozygote crosses did have greater postnatal and post-weaning mortality which was solely attributable to the loss of DAT −/− mice. No gross abnormalities or behavioral phenotypes were obvious in double KO mice beyond those that have already been reported for the single KO mice. All experiments were conducted in accordance with National Institutes of Health (USA) guidelines governing the use of animals in experimental research under protocols approved by the National Institute on Drug Abuse (USA) Intramural Research Program Animal Care and Use Committee (Protocol number 10-MNRB-07).

### Genotyping

Mice were genotyped by PCR for DAT and 5-HT_1B_ genotypes using two internal primers, one targeted at the NEO portion of the transgenic construct (KO primer) and one targeted at the WT gene (WT primer), and one external primer for each gene. PCR using Takara ExTaq or e2Tak DNA polymerase (Takara, Japan) was performed on tail DNA eluted after digestion overnight in Protease K. For DAT genotyping the forward primer (5′ GAC TTG CTG TGA CCT GGC TAG GAA C 3′) and the WT primer (5′ GCC TTG AGC TCT TAT CGG GCC TCC 3′) produced a 640 bp band, while the forward primer and the KO primer (5′ GCC TCT GTC CGC AGT TCA TTC AG 3′) produced a 900 bp band. For 5-HT_1B_ receptor genotyping the forward primer (5′ GAC TTG GTT CAC GTA CAC AG 3′) and the WT primer (5′ CCC ATC AGC ACC ATG TAC AC 3′) produced a 560 bp band, while the forward primer and the KO primer (5′ CTT CTA TCG CCT TCT TGA CG 3′) produced a 680 bp band. The PCR products were separated and visualized using gel electrophoresis for determination of individual genotypes.

### Locomotor Activity Testing

Locomotor activity was assessed in Plexiglas boxes (36×18×18 cm) using an automated Optovarimax system (Columbus Instruments, Columbus, OH, USA). The total distance traveled during each time bin was calculated from infrared beam-breaks by ATS software that localized the subject’s position in the apparatus (Columbus Instruments, Columbus, OH, USA). The locomotor activity monitors were placed in sound-attenuating chambers and all locomotor activity was assessed under dark conditions between 10 AM and 4 PM. The methods used to examine locomotor behavior are generally similar to our previous publications with DAT KO and DAT/SERT KO mice [11], [12]. Habituation, duration of testing and timing of drug injections was different for each experiment. These details are described below.

### Experiment 1: Basal and Cocaine-induced Locomotor Activity after an Extended Period of Habituation

Using a within subjects design, mice from all 9 DAT/5-HT_1B_ double KO genotype combinations (n = 9–11 per genotype) were placed in the locomotor test chambers and allowed to habituate for 3 hours prior to injection with cocaine (0.0, 5.0, 10.0 and 20.0 mg/kg s.c.). At least 48 hours separated each testing day. The order of doses was counter-balanced. Locomotor activity was assessed as described above. There was no evidence for locomotor sensitization when the order of testing was examined as a factor using analysis of variance (ANOVA; *data not presented*).

### Experiment 2: Cocaine-induced Locomotor Activity after a Brief Period of Habituation

To allow comparison to the results of previously reported locomotor studies in DAT KO mice that have used different periods of pre-injection habituation [10]–[13], locomotor activity was also assessed in all 9 genotypes of DAT/5-HT_1B_ double KO mice (N = 8–13 per genotype) after 30 minutes of habituation to the locomotor test chambers, using a within-subjects design after injections with 0 (saline vehicle) or 20 mg/kg s.c. cocaine. At least 48 hours separated each testing day and the dose order was counter-balanced.

### Experiment 3: Effects of the 5-HT antagonist SB224289 on locomotor activity in DAT KO and WT mice

Locomotor activity was assessed in DAT −/− and wildtype, DAT +/+ littermates (n = 10 mice per genotype) as previously described. Mice were injected with the 5-HT_1B_ antagonist SB224289 (0, 5, 10 or 20 mg/kg i.p.) prior to placement in locomotor activity chambers for 1 hour. A within-subjects design was used, at least 48 hours separated each testing day and the dose order was counter-balanced.

### Statistics

Statistical comparisons were made with ANOVA followed by Scheffe’s *post hoc* comparisons using Statview (SAS). Data are presented as mean ± SEM for each experimental group. In Experiment 1 there were substantial differences in basal activity across genotypes observed during the long habituation period. This difference in activity between genotypes did not equalize even by the end of this extended period of habituation. The habituation sessions were compared to each other to determine if the patterns observed in the first exposure were maintained or if there were differences in habituation resulting from DAT GENOTYPE or 5-HT_1B_ GENOTYPE or any other factors. Because the activity of the groups was not equalized by the end of the habituation period, cocaine-induced locomotion was analyzed in terms of difference scores of the summed activity over the two hours of testing post-cocaine minus the summed activity over the 2 hours of baseline activity immediately prior to cocaine injection. Since the short habituation data in Experiment 2 did not allow this correction, the difference in locomotor activity after saline injection was compared to locomotor activity after cocaine injection in the initial ANOVA, and then the time-course of cocaine and saline responses was examined in separate ANOVA for each genotype. In ANOVA, the between-subjects factors used were DAT GENOTYPE and 5-HT_1B_ GENOTYPE, along with the within-subjects factors TIME (for baseline locomotion), habituation SESSION (for pre-cocaine habituation sessions) and DOSE of cocaine or SB 224289, where appropriate.
